# Supplementary material for: A qualitative study on the reasons for solitary eating habits of older adults living with family
Source: PLoS One. 2020 Jun 8;15(6):e0234379. doi: 10.1371/journal.pone.0234379 (PMC7279577; doi:10.1371/journal.pone.0234379)
Supplement: S1 File — (DOC) [file pone.0234379.s001.doc]

**Interview guide**

**1. Basic information**

Age, family structure, living status, etc.

**2. Questions about eating**

- We found that you usually ate alone when you participated in the previous survey. Please tell me about the current situation of eating in details.
- From when do you eat alone? And why?
- How do you think of your eating alone behavior?
- Why don’t you eat together with your family member?
- Please tell me about your meal in details.

**3. Questions about functions**

- How is your oral condition?
- How is your body condition?
- How is your mental condition?
- How is your social condition?

**4. Other information**
